# Nurses' performance in using the APACHE score: Its effect on outcomes of patients with cardiac surgery

**DOI:** 10.25122/jml-2023-0059

**Published:** 2023-07

**Authors:** Asmaa Hany, Tahany Elsenousy, Asmaa Abd Elrahman, Shimaa Nabil

**Affiliations:** 1 Medical Surgical Intensive Care Unit, Specialized Hospital, Ain Shams University, Cairo, Egypt; 2 Critical Care and Emergency Nursing, Faculty of Nursing, Ain Shams University, Cairo, Egypt; 3 Medical Surgical Nursing, Faculty of Nursing, Ain Shams University, Cairo, Egypt

**Keywords:** APACHE Score, cardiac surgery, nurses performance, patient outcomes

## Abstract

The Acute Physiology and Chronic Health Evaluation (APACHE) score was developed to assess disease severity. Once organ failure develops, laboratory values and hemodynamic parameters should be monitored to assess the degree of impairment. This study aimed to assess nurses' performance regarding using the APACHE scoring system and its effect on the outcomes of patients with cardiac surgery. A quasi-experimental research design (one group pretest and post-test) was used to achieve the aim of this study. The convenience sample consisted of all available nurses (n=50) working in intensive care units of cardiac surgery at Ain Shams Specialized Hospital and in the Cardiovascular and Thoracic Academy affiliated with Ain Shams University hospitals. Additionally, a purposive sample of 130 patients with cardiac surgery was included in this study. Data was collected using nurses' self-administered questionnaires, observation checklists for nurses, attitude scales of nurses, patient assessment questionnaires, and nursing-sensitive patient outcomes measuring scales. The findings showed a highly statistically significant difference between the mean score of total nurses’ level of knowledge, practice, and attitude before and after the educational program. Also, a highly statistically significant difference was observed in the total score of patient outcomes before and after the implementation of the educational program. Implementing an educational program positively affected nurses’ performance; the APACHE score has an efficient and comparable discriminative ability to predict the outcomes of patients undergoing cardiac surgery. The study recommends conducting periodic training programs for nurses in cardiac surgery intensive care units to equip them with the latest knowledge and skills in using the APACHE scoring system to predict patient outcomes and complications within the first 24 hours.

## INTRODUCTION

The Acute Physiology and Chronic Health Evaluation (APACHE) score is a physiology-based index used to assess disease severity and predict the risk of death in hospitals for patients receiving treatment in critical care units. This tool consists of three parts: an acute physiology score (APS), a patient's chronic illness score, and an age score, which are assessed within 24 hours of admission to the intensive care unit (ICU) to define critically ill patients, estimate prognosis, and set a benchmark for the maintenance of standards of quality of care in the ICU [1].

Cardiac surgery is a major procedure that involves the heart structure to correct abnormalities in the heart muscle, including heart valves, coronary arteries, ventricular aneurysms, and septal defects. Cardiac surgeries are performed to restore blood flow and deliver oxygen to the heart muscle. There are two major types of cardiac surgeries: close-heart and open-heart surgery. Close heart surgery involves a small incision without using the heart-lung machine, and open-heart surgery, which requires opening the chest cavity and connecting the patient to a heart-lung machine involving the heart chambers [2].

In 2020, the Society of Thoracic Surgeons database estimated that coronary artery bypass graft (CABG) surgery constituted 55% of cardiac surgical procedures, while coronary artery bypass graft combined with one or more valve procedures accounted for another 9%. Alos, in 2020, 3658 heart transplants were performed in the United States [3]. Patients who undergo cardiac surgery are usually admitted to the critical care unit, and they are particularly prone to severe complications post-operatively due to pre-existing chronic conditions and the complex and prolonged techniques used during surgery. Caring for open heart surgery patients is complex and requires nurses with adequate knowledge and skills to perform the necessary tasks involved in the procedure [4].

Upon admission to the critical care unit in the first 15 minutes after surgery, nurses connect the patient to the cardiac monitor and document the rhythm of the heart, attach the arterial and pulmonary artery pressure lines to the monitor and measure cardiac index/output, note presence of vasoactive/inotropic infusion. The nurse should assess the breath sounds bilateral after connecting the ventilator to an endotracheal tube, attach capnography to the ventilator circuit to monitor end-tidal carbon dioxide, and evaluate waveform and value. Furthermore, they connect pulse oximetry devices to the patient, assess the consciousness level, and record oxygen saturation and waveforms [5].

Nurses' performance has a great impact on patients' outcomes. Nursing-sensitive indicators have been embraced as valid and reliable instruments due to their characteristics such as objective assessment, enhancement of clinical practice, evaluation of the quality of nursing care and performance, and help in decision making ability to provide appropriate and timely care. Nursing-sensitive indicators influence nursing care outcomes by defining the framework and processes of nursing care. In order to ensure good outcomes for patients with cardiac surgery, it is vital to equip nurses with the required knowledge and skills about tools used in assessing ICU patients, such as the APACHE score, to enhance the quality of care of cardiac surgery patients [6].

### Significance of the study

The use of the APACHE scoring system is considered important for patients with cardiac surgery because it could help nurses determine high-risk patients groups, assess hemodynamic instability based on the degree of physiological variables, predict mortality rate among patients within the first 24 hours of critical care unit admission, and measure patient outcomes. Therefore, this study was implemented to assess nurses’ performance in using the APACHE score for patients undergoing cardiac surgery to evaluate patient status, improve patient outcomes, and enhance the quality of nursing care provided to these patients.

### Aim of the study

This study aimed to assess nurses’ performance regarding the use of the APACHE score and its effect on outcomes of patients with cardiac surgery by:

1. Assessing nurses' knowledge, practice, and attitude regarding the use of the APACHE scoring system.
2. Developing and implementing an educational program regarding the APACHE scoring system for nurses caring for patients with cardiac surgery.
3. Evaluating the effect of implementing the APACHE scoring system program on patient outcomes following cardiac surgery.

## RESEARCH HYPOTHESES

1. Implementing the educational program would positively affect nurses’ knowledge regarding the APACHE scoring.
2. Implementing the educational program would positively affect nurses’ practice regarding the use of the APACHE scoring system.
3. Implementing the educational program would positively affect nurses’ attitude regarding the use APACHE scoring system.
4. Implementing the educational program would positively affect the outcomes of patients with cardiac surgery after using the APACHE scoring system.

## MATERIAL AND METHODS

### Research design and setting

This study used a quasi-experimental design with one group pre-test and post-test. The study was implemented in intensive care cardiac surgery units at Ain Shams Specialized Hospital and Cardiovascular and Thoracic Academy at Ain Shams University Hospitals, Cairo, Egypt.

### Participants

A convenience sample of fifty nurses who provided care for patients undergoing cardiac surgery in both intensive care units mentioned previously was included in this study. Additionally, a purposive sample of 130 patients undergoing cardiac surgery was included in this study. The sample size was calculated using a power analysis equation based on the number of patients admitted to the intensive care units (ICUs) during 2019/2020. The study aimed to achieve a test power of 80% and a confidence interval of 95%. The error margin was adjusted to 5% with a type I error (α) set at 0.05 and a type II error (B) at 0.20%. The power of the test was targeted at 0.80%.

### Data collection tools

Five data collection tools were used in this study:

#### Nurses’ knowledge questionnaire

The tool consisted of two parts. Part I assessed nurses’ demographic characteristics, such as age, gender, marital status, educational level, years of experience, and previous training. Part II assessed nurses' knowledge regarding the APACHE II scoring system. It was designed by the researchers in Arabic language based on previous related literature [7, 8]. This tool comprised 50 questions in various formats: twenty true/false questions, 25 multiple-choice questions, and 5 matching questions.

Scoring system

- The total score of the tool was 50 grades.
- The correct answer was given one grade.
- The incorrect answer was given zero.
- The total scores for nurses' knowledge were classified as satisfactory or unsatisfactory.
- ≥85% (≥40 degrees) was considered satisfactory.
- <85% (≤ 39 degrees) was considered an unsatisfactory level.

#### Nurses' observation checklist

The checklist was utilized to assess nurses’ practice related to monitoring physiological parameters, arterial blood gas sampling withdrawal via arterial line, and Glasgow coma scale evaluation. It was designed in English and adopted from other studies [9-11].

Scoring system

- The tool consisted of 122 steps.
- Each step was evaluated as correctly done, incorrectly done, or not done.
- The step correctly done was given one grade.
- The incorrectly/not done was given zero.
- The total score of this tool was categorized as satisfactory or unsatisfactory.
- ≥ 85% (≥103 degrees) was considered a satisfactory level of practice.
- <85% (<103 degrees) was considered unsatisfactory level of practice.

#### Attitude scale for nurses

This scale was used to assess the attitude of nurses toward using the APACHE II scoring system for patients with cardiac surgery. It was adapted from previous studies [12, 13], translated into Arabic, and then back-translated into English. It contained 25 items with responses on a Likert scale (zero-disagree, one-somehow agree, two-agree).

Scoring system

- The total score of all scale items is 50 grades.
- It was categorized as a positive attitude and a negative attitude.
- ≥85% (≥21 degrees) was considered a positive attitude.
- <85% (<21 degrees) was considered a negative attitude.

#### Patient assessment questionnaire

This tool included four parts. Part I assessed the demographic characteristics of patients, such as age, gender, marital status, educational level, occupation, past medical history, diagnosis, and type of cardiac surgery. Part II assessed patients' outcomes by assessing APACHE II physiological parameters. It was designed in English and adopted from other studies [14, 15]. It included 12 variables divided into a high and low abnormal range of the physiological parameters, which include temperature, mean of arterial blood pressure, heart rate, respiratory rate (on a ventilator or not), oxygenation record (A-aDO2 or PaO2), arterial pH or serum HCO3, sodium and potassium in the blood, serum creatinine, hematocrit, white blood cells and a score of Glasgow coma scale. Part 3 assessed patients' age, including five subcategories of age. Finally, part 4 assessed chronic health and included two subcategories of chronic health. Each category was given a score.

Scoring system of APACHE

- 0–4 = 4 % death rate
- 5–9 = 8% death rate
- 10–14 = 15% death rate
- 15–19 = 25% death rate
- 20–24 = 40% death rate
- 25–29 = 55% death rate
- 30–34 = 75% death rate
- Over 34 = ≥ 85% death rate

#### Nursing sensitive patient outcomes measuring scale

This scale was utilized to assess the bio-psychosocial outcomes of patients with cardiac surgery before and after conducting an educational program for nurses. It was designed in English and was adopted from previous studies [16, 17], consisting of 4 domains.

1. Physiological health outcomes

This domain focused on assessing the physiological and physical functioning of patients, such as the effectiveness of the cardiac pump, circulatory condition, vital signs, cardiac and peripheral tissue perfusion, coagulation profile, fluid and electrolyte balance, bowel elimination, nutritional status, medications response, control of pain and self-care.

Scoring system

- Not compromised= 0
- Moderate compromised= 1
- Extremely compromised= 2

2. Psychological health outcomes

The second domain assessed the psychological health of patients. It included two main items: (1) psychological well-being, which contained an assessment of self-esteem, body image, and identity, and (2) self-control, which assessed measures of anxiety control.

Scoring system

- Consistent demonstrate = zero
- Sometimes demonstrate = one
- Never demonstrate = two

3. Social health outcomes

This domain assessed social health. It included two parameters: (1) social interaction, which includes social involvement, social support, and role performance, and (2) psychosocial adaptation, which assessed health status acceptance and coping.

Scoring system

- Extensive = 0
- Moderate = 1
- None = 2

4. Perceived health assessment

The fourth domain assessed patients’ impressions of their health. It included one parameter only: (1) health and quality of life, including spiritual well-being.

Scoring system

- Not compromised = 0
- Moderately compromised = 1
- Extremely compromised = 2

### Validity and reliability

Face validity aims to assess the items to detect if the tools measure what they are expected to measure. In this study, the face and content validity of the tools were evaluated by seven experts from the medical-surgical nursing department of the faculty of nursing at Ain Shams University. The experts revised the content of the tools for clarity, relevance, simplicity, comprehensiveness, and applicability. Minor modifications were made. In this study, the reliability of the tools was tested using the alpha Cronbach test, a widely used method for assessing internal consistency. The Cronbach's alpha coefficient was calculated for each tool to assess the degree of correlation between its items. The obtained values were 0.801 for the nurses' knowledge assessment questionnaire, 0.736 for the observation checklist, 0.796 for the nurses' attitude scale, 0.767 for the patient assessment questionnaire, and 0.671 for the nurses' sensitive patient outcomes measuring scale.

### Pilot study

The pilot study was conducted on 10% (n=5) of nurses to assess the clarity and applicability of the tools and to determine the time needed to complete each tool. No changes were made, and the final form of the tools was developed. Subjects included in the pilot study were involved in the actual study.

### Fieldwork

The fieldwork included two phases:

#### Implementation phase

1. Data collection and the educational program were conducted over seventeen months, from January 2021 to May 2022.
2. Data was collected from the specified settings three days per week, during both morning and afternoon shifts.
3. The assessment of nurses' knowledge, practice, and attitude before the educational program took approximately ten weeks, from January 2021 until mid-March 2021.
4. The assessment of patients before the program began was carried out over four months, from January 2021 until April 2021.
5. The educational program was implemented over 10 weeks, from July 2021 until mid-September 2021. It included seven theory and three practical sessions, each lasting about one hour.
6. The first session of the theoretical part included the meaning, components, and high and low abnormal range of physiological variables of the APACHE II scoring system. The second session was concerned with using alveolar-arterial diffusion oxygen or A-A gradient equation or using PaO2 and age points of the APACHE II scoring system. The third session was focused on chronic health evaluation, calculation, interpretation, and use of the APACHE II scoring system. The fourth session included a Glasgow coma scale evaluation. The fifth session concerned the normal range of vital signs, laboratory investigation, and arterial blood gases. The sixth session included causes and symptoms of acid-base imbalance, and the seventh session focused on the maximum time allowed when arterial blood gases sample is withdrawn, analyzed, and interpreted.
7. Regarding the practical sessions, the eighth session concerned monitoring patients' physiological parameters following cardiac surgery. The ninth session focused on withdrawing arterial blood gas samples, and the tenth assessed the Glasgow coma scale.
8. After implementing the educational program, nurses' knowledge, practice, and attitude were assessed again. This evaluation process took 10 weeks, from mid-September 2021 until November 2021.
9. The assessment of patients after the educational program was also carried out, lasting four months from mid-September 2021 until mid-December 2021.
10. A follow-up test for nurses was conducted three months after the program, lasting 10 weeks from March 2022 until mid-May 2022.
11. Nurses' knowledge and attitude questionnaires were completed by the nurses themselves and took approximately 30-45 minutes each, while the observation checklist, patients' assessment questionnaire, and patients' outcomes were filled in by the researcher.

#### Evaluation phase

12. After the implementation of the program, all data collection tools were completed again immediately. The evaluation of the effect of the educational program was done by comparing the results of nurses' knowledge, practice, and attitude pre-, post-, and three months later using the same tools.
13. Patient assessment questionnaire and the patient outcomes measuring scale were filled in by the researcher two times (before and after conducting the educational program).

### Data analysis

The data collected from the study were analyzed using the Statistical Package for the Social Sciences (SPSS) version 23. Quantitative data were presented as mean, ranges, and standard deviation (SD). The comparison was done using paired sample t-test, inadequate samples t-test, and ANOVA test. The qualitative data were presented as numbers and percentages (%).

## RESULTS

Table 1 shows the demographic data of nurses and revealed that the mean age of nurses was 32.0±10.5. Most nurses (90%) were females, more than half (54%) were married, and two-fifths (42%) graduated from a technical health nursing institute. The mean years of experience for nurses in the study were 7.14±2.64 years, and none of them had attended any training courses on using the APACHE scoring system.

**Table 1:** Demographic characteristics of nurses (n=50)

Items	N	%
**Age**		
20-30 years	22	44.0
>30-40 years	12	24.0
>40-50 years	11	22.0
>50 years	5	10.0
**±SD** *^X^*	32.0±10.5	
**Gender**		
Male	5	10
Female	45	90
**Marital status**		
Single	23	46
Married	27	54
**Qualifications**		
Nursing diploma	17	34
Technical health nursing institute	21	42
Bachelor of Nursing	12	24
**Years of experience in cardiac surgery ICU**		
Less than 5 years	23	46
Between 5 to 10 years	9	18
More than 10 years	18	36
**±SD** *^X^*	7.14±2.64	
**Attended training courses regarding the use of the APACHE scoring system**		
Yes	0	0
No	50	100

Table 2 presents the statistically significant differences between the mean scores of nurses’ total level of knowledge regarding the APACHE II scoring system, Glasgow coma scale, arterial blood gases, and normal range of laboratory values pre-and post-implementation of the educational program, as well as pre and follow up (p-value <0.001). However, there was no statistically significant difference between the mean score of nurses’ total knowledge between the post-implementation and follow-up phases of the educational program (p= 0.384).

**Table 2:** Difference between the mean score of nurses’ knowledge regarding components of the APACHE II scoring system pre, post, and follow-up implementation of the educational program (n=50)

Nurses’ level of knowledge about components of APACHE II score	Pre-program	Post-program	Follow-up	Pre & Post	Pre & follow-up	Post & follow-up
	mean± SD	mean± SD	mean± SD	p-value	p-value	p-value
APACHE II scoring system definition, component, physiological parameters, age points, and chronic health points	12.64±3.53	20.02±2.58	19.12±2.46	<0.001**	<0.001**	0.078
Glasgow coma scale	5.44±1.97	7.68±1.04	7.42±1.23	<0.001**	<0.001**	0.256
Arterial blood gases	5.82±2.82	9.20±2.01	9.36±2.25	<0.001**	<0.001**	0.708
Normal range of laboratory values in APACHE II score	3.64±1.48	4.68±0.82	4.80±0.70	<0.001**	<0.001**	0.433
**Total score of knowledge**	27.54±7.37	41.58±4.93	40.70±5.14	<0.001**	<0.001**	0.384

Paired Sample t-test, p-value >0.05 not significant; p-value <0.001 highly significant**

Regarding nurses' practice, there was a highly statistically significant difference between the mean score of nurses’ practice in relation to physiological parameters of the APACHE II system, arterial blood gases sampling, and Glasgow coma scale evaluation pre and post-implementation of the educational program, and between pre and follow-up assessment (p-value<0.001) (Table 3). However, there was no statistically significant difference between the post-implementation and follow-up mean total practice scores among nurses after the educational program (p= 0.122).

**Table 3:** Difference between the mean score of nurses’ practice about using of APACHE II scoring system pre, post, and follow-up implementation of educational program (n=50)

Total score of nurses’ practice	Pre-program	Post-program	Follow-up	Pre & Post	Pre & follow-up	Post & follow-up
	mean± SD	mean± SD	mean± SD	p-value	p-value	p-value
Monitor the physiological parameters of the APACHE II scoring system	33.73±9.72	55.42±9.48	52.36±15.68	<0.001**	<0.001**	0.301
Arterial blood gas sampling via arterial line	85.20±21.11	119.26±16.94	115.40±32.60	<0.001**	<0.001**	0.127
Assessment of Glasgow Coma scale	12.02±3.25	20.88±1.59	19.43±2.84	<0.001**	<0.001**	0.109
**Total score of nurses’ practice**	130.95±32.57	195.56±27.85	187.19±50.90	<0.001**	<0.001**	0.122

Paired sample t-test, p-value >0.05 not significant; p-value <0.001 highly significant**

Moreover, there was a highly statistically significant difference between the mean score of nurses’ attitudes regarding the use of the APACHE II scoring system before and after implementing the educational program (39.10±6.54 and 44.26±3.26 respectively), as well as between the mean score of the pre and follow-up assessment (p-value<0.001) (Table 4). There was no statistically significant difference in the post-implementation and follow-up mean scores of nurses' attitudes after the educational program (p-value=0.413).

**Table 4:** Difference between the mean score of nurses’ attitude regarding the use of the APACHE II scoring system pre, post, and follow-up implementation of the educational program (n=50)

Total score of nurses’ attitude	Pre-program	Post-program	Follow-up	Pre & Post	Pre & follow-up	Post & follow-up
	mean± SD	mean± SD	mean± SD	p-value	p-value	p-value
Total score of attitude	39.10±6.54	44.26±3.26	43.78±2.53	<0.001**	<0.001**	0.413
Total % score of attitude	78.20±13.08	88.52±6.53	87.56±5.05	<0.001**	<0.001**	0.413

Paired sample t-test, p-value >0.05 non-significant; p-value <0.001 highly significant**

The study found that before the educational program implementation, 36.2% of patients had an APACHE II score ranging from 10-14, which predicts a 15% death rate. However, after the implementation of the educational program, a significant improvement was observed, with 51.5% of patients obtaining an APACHE II score ranging from 5-9, predicting a lower (8%) death rate (Table 5). In addition, there was a highly statistically significant difference between the total APACHE II scoring system before and after the implementation of the educational program (p-value <0.001).

**Table 5:** Frequency and percentage distribution of patients at risk for death based on APACHE II scoring system pre-and post-implementation of the educational program (n=130)

Interpretation of APACHE II Score	Pre		Post		Chi-square test	
	No.	%	No.	%	x^2^	p-value
Score 0-4 = 4% Death Rate	5	3.8	21	16.2	31.192	<0.001**
Score 5-9 = 8% Death Rate	34	26.2	67	51.5		
Score 10-14 = 15% Death Rate	47	36.2	29	22.3		
Score 15-19 = 25% Death Rate	33	25.3	10	7.7		
Score 20-24 = 40% Death Rate	7	5.4	3	2.3		
Score 25-29 = 55% Death Rate	4	3.1	0	0.0		
Score 30-34 = 75% Death Rate	0	0.0	0	0.0		
Score Over 34 = ≥ 85% Death Rate	0	0.0	0	0.0		
Total	130	100.0	130	100.0		

Chi-square test; not significant (p>0.05), **highly significant (p<0.001)

Furthermore, there was a highly statistically significant difference between the total score of the physiological, psychological, social, and spiritual health of patients before and after the implementation of the educational program (p-value <0.001) (Table 6). The total score of nursing-sensitive patient outcomes also demonstrated a highly statistically significant difference before and after the implementation of the educational program, with a p-value of less than 0.001.

**Table 6:** Frequency and percentage distribution of the total nursing sensitive patients’ outcomes among the studied patients pre-and post-implementation of the program (n=130)

Nursing Sensitive Patient Outcome	Pre-program (n=130)		Post-program (n=130)		Chi-square test	
	No.	%	No.	%	x^2^	p-value
**Physiological health**						
Not Compromised	62	47.7	94	72.3	26.843	<0.001**
Moderate Compromised	50	38.5	36	27.7		
Severe Compromised	18	13.8	0	0.0		
**Psychological health**						
Not Compromised	81	62.3	107	82.3	12.005	<0.001**
Moderate Compromised	49	37.7	23	17.7		
Severe Compromised	0	0.0	0	0.0		
**Social Health**						
Not Compromised	73	56.2	101	77.7	12.662	<0.001**
Moderate Compromised	57	43.8	29	22.3		
Severe Compromised	0	0.0	0	0.0		
**Health and quality of life: Spiritual well-being**						
Not Compromised	81	62.3	117	90.0	25.945	<0.001**
Moderate Compromised	49	37.7	13	10.0		
Severe Compromised	0	0.0	0	0.0		
**Total Nursing Sensitive Patient Outcome**						
Not Compromised	73	56.2	104	80.0	19.096	<0.001**
Moderate Compromised	52	40.0	26	20.0		
Severe Compromised	5	3.8	0	0.0		

Chi-square test, p-value >0.05 not significant; *p-value <0.05 significant; **p-value <0.001 highly significant

## DISCUSSION

Regarding the demographic characteristics of the participating nurses, the mean age was 32.0±10.5, with an age range of 20 to >50 years old. The majority of nurses were females, and more than half were married. These findings align with a previous study [18], where a similar age range of 20 to 40 years was observed, and all nurses were females, with a majority being married.

In terms of educational level, 42% of nurses graduated from the technical health nursing institute, which is in line with the findings of Reisdorfer *et al*. [19], who stated that 34% of nurses graduated from the technical health nursing institute in their study. Additionally, the mean years of nursing experience were 7.14±2.64, similar to Hameed and Dawood [20], who reported that 51.9% of nurses working in cardiac surgery ICU had 1-2 years of experience.

The study found that none of the nurses had attended any training courses related to using the APACHE scoring system. This finding is incongruent with Adams *et al*. (2020) [21], who stated that 97% of nurses attended training courses on using the APACHE II scoring system in their study. Regarding nurses’ knowledge, the results of the current study reported highly statistically significant differences between the mean score of nurses’ knowledge regarding APACHE II scoring system content pre-and post-implementation of the educational program and between the pre and follow-up assessments. This may be attributed to the content of the program that was designed according to the needs of nurses, the clear and straightforward language used during the explanation of the content, and the availability of the researcher in the cardiac surgery intensive care units during the working hours of the staff to clarify any misunderstandings.

The findings of our study are in line with previous research conducted by Hussein *et al*. (2021) and Lim (2014), which demonstrated significant improvements in nurses' knowledge after the implementation of specialized educational programs focused on the APACHE II scoring system and coronary artery bypass graft surgery, respectively [22, 23].

The significant improvement in nurses' practice observed in our study can be attributed to the focused and comprehensive educational program that addressed specific areas of deficiency in their understanding and skills. Before the program, there was a lack of understanding among nurses regarding key components of the APACHE II scoring system, such as the calculation of oxygenation and the use of double points for serum creatinine in cases of acute renal failure. Additionally, some nurses faced challenges in interpreting arterial blood gases accurately and conducting thorough Glasgow coma scale assessments. This lack of proficiency may have been exacerbated by the absence of prior training courses related to APACHE II scoring or cardiac surgery. However, implementing the educational program effectively addressed these knowledge gaps and provided nurses with the necessary skills and knowledge to confidently and accurately monitor physiological parameters, perform arterial blood gas sampling, and assess patients using the Glasgow coma scale.

This finding is consistent with the results of Eltayed *et al*. in their study on invasive hemodynamic monitoring training for ICU nurses in a cardiac center in Sudan [24]. Eltayed *et al*. observed a substantial improvement from 34% to 80.9% in ICU nurses' practice regarding invasive hemodynamic monitoring after the interventional program, which was considered a satisfactory practice level regarding invasive hemodynamic monitoring.

Our study's results on nurses' attitudes toward the use of the APACHE II scoring system align with the findings of Nesami *et al*. (2022) in their study on the effect of education on the knowledge and attitude of intensive care unit (ICU) staff toward the use of predictive disease severity scoring systems [25]. Both studies observed a highly significant difference in nurses' attitudes pre-and post-implementation of educational programs.

Regarding patients at risk for death, according to the APACHE II scoring system, the results showed that more than one-third of patients had an APACHE II score ranging from 10 to 14, which predicts a 15% death rate before the implementation of the educational program. In contrast, more than half of the patients had scores ranging from 5 to 9, which predicts an 8% death rate after implementation of the educational program. Moreover, there was a highly statistically significant difference between the risk of death according to the APACHE II scoring system pre-and post-implementation of the educational program. The prediction of the mortality rate according to the APACHE II scoring system among patients pre-and post-implementation of the educational program was concurrent with the actual mortality rate documented among the studied group of patients. These results agree with Bahtouee *et al*. (2019) [26], who mentioned that there was a significant relationship between different levels of APACHE II score and patient outcomes so that approximately 80% of patients with APACHE II scores less than 15 survived, while more than 80% of patients with APACHE II ≥15 died. Also, Elhamshary *et al*. (2019) [27] stated that the APACHE II scoring system showed high power in predicting mortality rates after cardiac surgery and is the best tool for risk classification among CABG patients.

The findings of our study regarding total nursing-sensitive patient outcomes demonstrate a significant improvement in patients’ physiological, psychological, social, and spiritual health after the implementation of the educational program. Furthermore, a majority of patients did not experience deterioration after the program's implementation, with highly statistically significant differences observed between pre- and post-implementation assessments. This may be attributed to the effect of educational program on nurses' performance, which reflects directly on patients' outcomes. These findings are consistent with the study conducted by Ebada *et al*. (2017) [28], which also reported improved nursing-sensitive patient outcomes after implementing self-care guidelines, with a statistically significant difference between pre-and post-implementation.

## RECOMMENDATIONS

The study results underscore the importance of implementing strategies to enhance nurses' knowledge and practice in cardiac surgery intensive care units, particularly concerning the use of the APACHE II scoring system. To achieve this, we recommend conducting periodic training sessions on advanced care for nurses in these units. These training sessions should focus on updating their knowledge and practices of the APACHE II scoring system, enabling them to effectively predict complications among patients undergoing cardiac surgery.

Furthermore, developing a comprehensive procedure book specifically tailored to cardiac surgery patients' care during the critical first 24 hours would be beneficial. This book should include detailed guidelines for nurses on performing hemodynamic assessments, accurately monitoring arterial blood gases through arterial lines, and conducting Glasgow coma scale evaluations. This resource would be a practical and accessible reference, ensuring that nurses are well-equipped to deliver efficient, high-quality patient care during this crucial post-operative period. Additionally, it is essential to institutionalize the use of the APACHE II scoring system in cardiac surgery critical care units. This can be accomplished through regular clinical meetings, workshops, seminars, and ongoing training programs.

## CONCLUSION

The results of the current study supported the first, second, and third hypotheses, where a highly statistically significant difference was found between the mean score of nurses’ total knowledge, practice, and attitude before and after the implementation of the educational program. Furthermore, our study also revealed a significant difference in the risk of death assessed by the APACHE II scoring system before and after the implementation of the educational program, which supported the fourth hypothesis. Moreover, a statistically significant difference was reported between the total score of the physiological, psychological, social, and spiritual health of patients' outcomes pre-and post-implementation of the educational program, also supporting the fourth hypothesis.

## Personal thanks

The authors thank nurses and patients who agreed to participate in this study. Their valuable time and cooperation in completing the questionnaire and attending the training program are greatly appreciated
